# Venom-Derived Peptide Modulators of Cation-Selective Channels: Friend, Foe or Frenemy

**DOI:** 10.3389/fphar.2019.00058

**Published:** 2019-02-26

**Authors:** Saumya Bajaj, Jingyao Han

**Affiliations:** Lee Kong Chian School of Medicine, Nanyang Technological University, Singapore, Singapore

**Keywords:** ion channel, venom, toxin peptides, animal toxin, ion channel pharmacology

## Abstract

Ion channels play a key role in our body to regulate homeostasis and conduct electrical signals. With the help of advances in structural biology, as well as the discovery of numerous channel modulators derived from animal toxins, we are moving toward a better understanding of the function and mode of action of ion channels. Their ubiquitous tissue distribution and the physiological relevancies of their opening and closing suggest that cation channels are particularly attractive drug targets, and years of research has revealed a variety of natural toxins that bind to these channels and alter their function. In this review, we provide an introductory overview of the major cation ion channels: potassium channels, sodium channels and calcium channels, describe their venom-derived peptide modulators, and how these peptides provide great research and therapeutic value to both basic and translational medical research.

## Introduction

Cone snails whose shells are coveted for their elaborate patterns, yellow dart frogs measuring just a few centimeters long, and transparent bell-shaped jellyfish with delicate tentacles might all seem unlikely candidates, but they are among the deadliest animals in the world. Like the more obvious perilous creatures – venomous snakes, spiders and scorpions – these animals release toxins that dramatically modulate the activity of various targets including ion channels, thereby affecting cellular communication and disrupting normal biochemical and physiological processes in prey or predator.

Animal venom is a complex mixture of various components – inorganic salts, organic molecules like alkaloids, proteins and peptides (King, 2011). While this concoction enables a multi-pronged attack upon the target organism, it has also led to an entire collection of bio-active compounds being available to researchers for probing the structural and functional properties of their molecular targets. Since ion channels play an essential role in neuronal signaling and muscle contractions, it is unsurprising that many venom toxins have evolved to block or modulate the function of ion channels (Dutertre and Lewis, 2010). Not only have venom-derived peptides been used extensively in probing ion channels, the understanding of the mechanism of this interaction has also led to the development of venom-based therapeutics targeting various ion channels. In fact, the recognition of animal venom having medicinal benefits is not a recent phenomenon. Venom from various animals had been used as medicines for centuries, in civilizations all over the world (Bhattacharjee and Bhattacharyya, 2014; Utkin, 2015).

**FIGURE 1:**
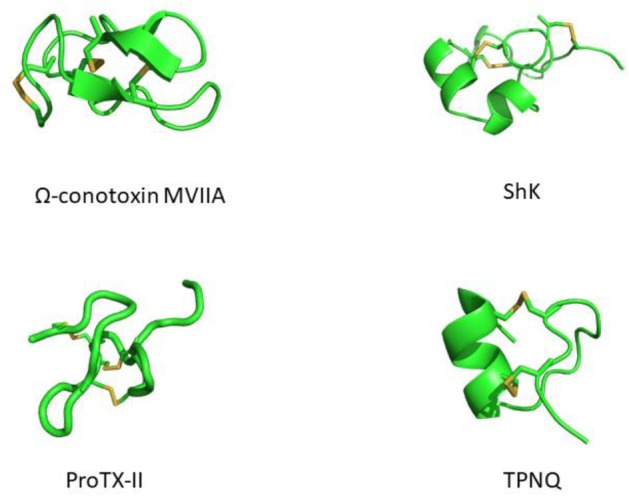
Structures of venom-derived peptide toxins – clockwise from top-left: ω-conotoxin MVIIA (PDB: 1MVI), ShK (PDB: 1ROO), TPNQ (PDB: 1TER), and ProTX-II (PDB: 2N9T). Disulfide linkages are shown in yellow.

Modern medicine has shown conclusively that venoms contain compounds with therapeutic potential. Many of these have been isolated, analyzed for structure and function, and have served as scaffolds for the development of various drugs. Venom peptides have evolved to be highly stable, being able to withstand degradation by proteolytic enzymes in the foreign environment they are injected into and in the venom itself. This stability is conferred by one or more disulfide bridges (Figure 1). While the peptides mutate into more potent and/or selective variants, the structurally important cysteines tend to be highly conserved. Cystine-stabilized α/β fold, inhibitor cystine knot (ICK, or knottin) and the three-finger toxin motif are all highly prevalent motifs in these peptides (Undheim et al., 2016).

This mini-review briefly describes exemplar peptides derived from animal venom, which have been used to probe the structure and function of voltage-activated cation channels, as well as are being developed as potential therapeutics (listed in Table 1). Here, we describe ion channels that are selectively permeable to potassium, calcium, and sodium ions.

**Table 1:** Venom-derived peptide modulators of cation channels.

Channel	Toxin	Species	IC_50_/*K*_d_	Reference
Kir1.1	Lq2 (α-KTx1.2)	*Leiurus quinquestriatus*	410 nM	Lu and MacKinnon, 1997
	δ-DTX	*Dendroaspis angusticeps*	150 nM	Imredy et al., 1998
	Tertiapin (TPN)	Apis mellifera	2 nM	Jin and Lu, 1998
Kir3.1/Kir3.4	Tertiapin (TPN)	Apis mellifera	8 nM	Jin and Lu, 1998
K_v_1.1	α-DTx	*Dendroaspis angusticeps*	20 nM	Grissmer et al., 1994
	DTx K (toxin I)	*Dendroaspis polylepis*	50 nM	Robertson and Owen, 1993
	α-KTx 2.2 (margatoxin)	*Centruroides margaritatus*	4.2 nM	Bartok et al., 2014
	α-KTx 2.5 (hongotoxin)	*Centruroides limbatus*	31 pM	Koschak et al., 1998
	α-KTx 3.13	*Mesobuthus eupeus*	203 pM	Gao et al., 2010
	ShK	*Stichodactyla helianthus*	16 pM	Kalman et al., 1998
K_v_1.2	α-DTx	*Dendroaspis angusticeps*	17 nM	Grissmer et al., 1994
	α-KTx 1.1 (Charybdotoxin)	*Leiurus quinquestriatus hebraeus*	9 nM	Takacs et al., 2009
	α-KTx 10.1 (Cobatoxin-1)	*Centruroides noxius*	27 nM	Jouirou et al., 2004
	α-KTx 2.1 (Noxiustoxin)	*Centruroides noxius*	2 nM	Grissmer et al., 1994
	α-KTx 2.2 (margatoxin)	*Centruroides margaritatus*	6 pM	Bartok et al., 2014
	α-KTx 2.5 (hongotoxin)	*Centruroides limbatus*	0.17 nM	Koschak et al., 1998
	α-KTx 3.6 (mesomartoxin)	*Mesobuthus martensii*	15 nM	Wang et al., 2015
	α-KTx 6.4	*Pandinus imperator*	8 pM	Sarrah et al., 2003
	α-KTx-6.2 (Maurotoxin)	*Scorpio maurus palmatus*	0.8 nM	Ryadh et al., 2018
	α-KTx-6.21 (Urotoxin)	*Urodacus yaschenkoi*	160 pM	Luna-Ramírez et al., 2014
	ShK	*Stichodactyla helianthus*	9 nM	Kalman et al., 1998
	BscTx1	*Bunodosoma caissarum*	30 pM	Orts et al., 2013
K_v_1.3	α-KTx 6.12 (Anuroctoxin)	*Anuroctonus phaiodactylus*	0.73 nM	Bagdáany et al., 2005
	α-KTx 3.12 (Aam-KTX)	*Androctonus amoreuxi*	1.1 nM	Abbas et al., 2008
	α-KTx 2.1 (Noxiustoxin)	*Centruroides noxius*	1 nM	Grissmer et al., 1994
	α-KTx 2.2 (margatoxin)	*Centruroides margaritatus*	11 pM	Bartok et al., 2014
	α-KTx 2.5 (hongotoxin)	*Centruroides limbatus*	86 nM	Koschak et al., 1998
	α-KTx 6.15 (Hemitoxin)	*Hemiscorpius lepturus*	2 nM	Najet et al., 2008
	α-KTx 6.3 (Neurotoxin)	*Heterometrus spinifer*	12 pM	Lebrun et al., 1997
	α-KTx 3.2 (Agitoxin-2)	*Leiurus quinquestriatus hebraeus*	200 pM	Garcia et al., 1994
	α-KTx 12.5 (LmKTx10)	*Lychas mucronatus*	28 nM	Liu et al., 2009
	α-KTx 3.11	*Odonthobuthus doriae*	7.2 nM	Abdel-Mottaleb et al., 2008
	α-KTx3.7	*Orthochirus scrobiculosus*	14 pM	Mouhat et al., 2005
	α-KTx 23.1 (Vm24)	*Vaejovis mexicanus smithi*	2.9 pM	Varga et al., 2012
	ShK	*Stichodactyla helianthus*	11 pM	Kalman et al., 1998
K_v_1.6	α-DTx	*Dendroaspis angusticeps*	9 nM	Swanson et al., 1990
	α-KTx 1.1 (Charybdotoxin)	*Leiurus quinquestriatus hebraeus*	22 nM	Garcia et al., 1994
	α-KTx 3.2 (Agitoxin-2)	*Leiurus quinquestriatus hebraeus*	37 pM	Garcia et al., 1994
	ShK	*Stichodactyla helianthus*	165 pM	Kalman et al., 1998
	BcSTx1/BcSTx2	*Bunodosoma caissarum*	1.3 nM/7.7 nM	Orts et al., 2013
K_v_2.1	HaTx1 (Hanatoxin)	*Grammostola spatulata*	42 nM	Swartz and MacKinnon, 1995
	JZTX-III/JZTX-XI	*Chilobrachys jingzhao*	710 nM/390 nM	Tao et al., 2013, 2016
	ScTx1	*Stromatopelma calceata*	12.7 nM	Escoubas et al., 2002
K_v_2.2	ScTx1	*Stromatopelma calceata*	21.4 nM	Escoubas et al., 2002
K_v_3.2	ShK	*Stichodactyla helianthus*	6 nM	Yan et al., 2005
K_v_3.4	BDS-I/BDS-II	*Anemonia sulcata*	47 nM/56 nM	Diochot et al., 1998
K_v_4.1	JZTX-XII	*Chilobrachys jingzhao*	363 nM	Yuan et al., 2007
K_v_4.2	PaTx1/PaTx2	*Phrixotrichus auratus*	5 nM/34 nM	Diochot et al., 1999
	ScTx1	*Stromatopelma calceata*	1.2 nM	Escoubas et al., 2002
	TsTx-Kβ (Ts8)	*Tityus serrulatus*	652 nM	Pucca et al., 2016
	HpTx3 (Heteropodatoxin)	*Heteropoda venatoria*	67 nM	Sanguinetti et al., 1997
	JZTX-V	*Chilobrachys jingzhao*	604.2 nM	Zeng et al., 2007
K_v_4.3	PaTx1/PaTx2	*Phrixotrichus auratus*	28 nM/71 nM	Diochot et al., 1999
	SNX-482	*Hysterocrates gigas*	3 nM	Kimm and Bean, 2014
K_Ca_1.1	α-KTx1.1 (Charybdotoxin)	*Leiurus quinquestriatus*	2.9 nM	Rauer et al., 2000
	α-KTx 1.3 (Iberiotoxin)	*Mesobuthus tamulus*	1.7 nM	Candia et al., 1992
	α-KTx 1.5 (BmTx1)	*Buthus martensi Karsch*	0.6 nM	Romi-Lebrun et al., 1997
	α-KTx 1.6 (BmTx2)	*Buthus martensi Karsch*	0.3 nM	Romi-Lebrun et al., 1997
	α-KTx 1.11 (Slotoxin)	*Centruroides noxius*	1.5 nM	Garcia-Valdes et al., 2001
	α-KTx 3.1 (Kaliotoxin)	*Androctonus mauretanicus*	20 nM	Crest et al., 1992
	α-KTx 3.5 (Kaliotoxin2)	*Androctonus australis*	135 nM	Crest et al., 1992
	α-KTx 12.1 (Butantoxin) / TsTX-IV	*Tityus serrulatus*	50 nM	Novello et al., 1999
	α-KTx (BmP09)	*Buthus martensi Karsch*	27 nM	Yao et al., 2005
	Natrin	*Naja naja atra*	34.4 nM	Wang et al., 2005
K_Ca_2.1	α-KTx 5.1 (Leiurotoxin I/scyllatoxin)	*Leiurus quinquestriatus hebraeus*	325 nM	Castle and Strong, 1986
	Tamapin	*Mesobuthus tamulus*	32 nM	Pedarzani et al., 2002
	Apamin	*Apis mellifera*	8 nM	Hugues et al., 1982
K_Ca_2.2	α-KTx 5.1 (Leiurotoxin I/scyllatoxin)	*Leiurus quinquestriatus hebraeus*	200 pM	Castle and Strong, 1986
	PO5	*Androctonus mauretanicus*	22 nM	Zerrouk et al., 1993
	Tamapin	*Mesobuthus tamulus*	24 pM	Pedarzani et al., 2002
	Apamin	*Apis mellifera*	30-200 pM	Hugues et al., 1982
	TsK	*Tityus serrulatus*	80 nM	Lecomte et al., 1999
K_Ca_2.3	α-KTx 5.1 (Leiurotoxin I/scyllatoxin)	*Leiurus quinquestriatus hebraeus*	1.1 nM	Castle and Strong, 1986
	PO5	*Androctonus mauretanicus*	25 nM	Zerrouk et al., 1993
	Tamapin	*Mesobuthus tamulus*	1.7 nM	Pedarzani et al., 2002
	Apamin	*Apis mellifera*	10 nM	Hugues et al., 1982
	TsK	*Tityus serrulatus*	197 nM	Lecomte et al., 1999
K_Ca_3.1	α-KTx 1.1 (Charybdotoxin)	*Leiurus quinquestriatus hebraeus*	5 nM	Ghanshani et al., 2000; Rauer et al., 2000
	α-KTx 6.2 (Maurotoxin)	*Maurus palmatus*	1 nM	Castle, 2003
	Margatoxin	*Centruroides margaritatus*	459 nM	Garcia-Calvo et al., 1993
	α-KTx 3.7 (OSK1)	*Orthochirus scrobiculosus*	225 nM	Mouhat et al., 2005
	ShK	*Stichodactyla helianthus*	30 nM	Pennington et al., 1995
	BgK	*Bunodosoma granulifera*	172 nM	Cotton et al., 1997
Na_v_1.1	MeuNaTxα-12	*Mesobuthus eupeus*	0.91 μM	Zhu et al., 2012
	MeuNaTxα-13	*Mesobuthus eupeus*	2.5 μM	Zhu et al., 2012
	ATX-II	*Anemonia sulcata*	6 nM	Chahine et al., 1996; Oliveira et al., 2004
	Cangitoxin-II; CGTX-II	*Bunodosoma cangicum*	0.165 μM	Zaharenko et al., 2012
	Bc-III	*Bunodosoma caissarum*	300 nM	Oliveira et al., 2004
	AFT-II	*Anthopleura fuscoviridis*	391 nM	Oliveira et al., 2004
	GVIIJ_SSG_	*Conus geographus*	11 nM	Gajewiak et al., 2014
	μ-Conotoxin BuIIIA	*Conus bullatus*	0.35 μM	Wilson et al., 2011
Na_v_1.2	Huwentoxin IV	*Haplopelma schmidti*	150 nM	Minassian et al., 2013
	ATX-II	*Anemonia sulcata*	41 nM	Oliveira et al., 2004
	Bc-III	*Bunodosoma caissarum*	1449 nM	Oliveira et al., 2004
	AFT-II	*Anthopleura fuscoviridis*	1998 nM	Oliveira et al., 2004
	Lqh-2	*Leiurus quinquestriatus hebraeus*	1.8 nM	Chen et al., 2002
	PnTx1	*Phoneutria nigriventer*	33.7 nM	Silva et al., 2012
	Phrixotoxin 3 (PaurTx3)	*Phrixotrichus auratus*	0.6 nM	Bosmans et al., 2006
	ProTx-III	*Thrixopelma pruriens*	0.3 μM	Cardoso et al., 2015
	Hainantoxin-IV	*Ornithoctonus hainana*	36 nM	Liu et al., 2003
	GrTx1	*Grammostola rosea*	0.23 μM	Redaelli et al., 2010
	GVIIJ_SSG_	*Conus geographus*	11 nM	Gajewiak et al., 2014
	μ-conotoxin TIIIA	*Conus tulipa*	0.045 μM	Wilson et al., 2011
	μ-conotoxin SIIIA	*Conus striatus*	0.05 μM	Wilson et al., 2011
	μ-conotoxin KIIIA	*Conus kinoshitai*	0.003 μM	Wilson et al., 2011
	μ-conotoxin MIIIA	*Conus magus*	0.45 μM	Wilson et al., 2011
	μ-conotoxin BuIIIA	*Conus bullatus*	0.012 μM	Wilson et al., 2011
Na_v_1.3	AFT-II	*Anthopleura fuscoviridis*	460 nM	Oliveira et al., 2004
	ATX-II	*Anemonia sulcata*	759 nM	Oliveira et al., 2004
	Bc-III	*Bunodosoma caissarum*	1458 nM	Oliveira et al., 2004
	ProTx-III	*Thrixopelma pruriens*	0.9 μM	Cardoso et al., 2015
	Hainantoxin-IV	*Ornithoctonus hainana*	375 nM	Liu et al., 2003
	GrTx1	*Grammostola rosea spider*	0.77 μM	Redaelli et al., 2010
	GVIIJ_SSG_	*Conus geographus*	15 nM	Gajewiak et al., 2014
	μ-conotoxin BuIIIA	*Conus bullatus*	0.35 μM	Wilson et al., 2011
Na_v_1.4	AFT-II	*Anthopleura fuscoviridis*	31 nM	Oliveira et al., 2004
	ATX-II	*Anemonia sulcata*	109 nM	Oliveira et al., 2004
	Bc-III	*Bunodosoma caissarum*	821 nM	Oliveira et al., 2004
	MrVIB (μO-Conotoxin)	*Conus marmoreus*	222 nM	Zorn et al., 2006
	MfVIA (μO-Conotoxin)	*Conus magnificus*	81 nM	Vetter et al., 2012
	GrTx1	*Grammostola rosea*	1.3 μM	Redaelli et al., 2010
	GVIIJ_SSG_	*Conus geographus*	47 nM	Gajewiak et al., 2014
	μ-conotoxin TIIIA	*Conus tulipa*	0.005 μM	Wilson et al., 2011
	μ-conotoxin SIIIA	*Conus striatus*	0.13 μM	Wilson et al., 2011
	μ-conotoxin MIIIA	*Conus magus*	0.33 μM	Wilson et al., 2011
	μ-conotoxin BuIIIA	*Conus bullatus*	0.012 μM	Wilson et al., 2011
Na_v_1.5	ProTx-II	*Thrixopelma pruriens*	79 nM	Middleton et al., 2002
	ATX-II	*Anemonia sulcata*	49 nM	Oliveira et al., 2004
	AFT-II	*Anthopleura fuscoviridis*	62.5 nM	Oliveira et al., 2004
	Bc-III	*Bunodosoma caissarum*	307 nM	Oliveira et al., 2004
	CGTX-II	*Bunodosoma cangicum*	50 nM	Zaharenko et al., 2012
Na_v_1.6	ATX-II	*Anemonia sulcata*	180 nM	Oliveira et al., 2004
	AFT-II	*Anthopleura fuscoviridis*	300 nM	Oliveira et al., 2004
	Bc-III	*Bunodosoma caissarum*	900 nM	Oliveira et al., 2004
	ProTx-II	*Thrixopelma pruriens*	47 nM	Maertens et al., 2006
	CGTX-II	*Bunodosoma cangicum*	50 nM	Zaharenko et al., 2012
	ProTx-III	*Thrixopelma pruriens*	0.29 μM	Cardoso et al., 2015
	GrTx1	*Grammostola rosea spider*	0.63 μM	Redaelli et al., 2010
Na_v_1.7	ProTx-I	*Thrixopelma pruriens*	51 nM	Middleton et al., 2002
	ProTx-II	*Thrixopelma pruriens*	300 pM	Schmalhofer et al., 2008
	ProTx-III	*Thrixopelma pruriens*	2.1 nM	Cardoso et al., 2015
	Lqh-2	*Leiurus quinquestriatus hebraeus*	32 nM	Chen et al., 2002
	Lqh-3	*Leiurus quinquestriatus hebraeus*	13.6 nM	Chen et al., 2002
	GpTx-1	*Grammostola porteri*	10 nM	Murray et al., 2015
	μ-SLPTX-Ssm6a	*Scolopendra subspinipes mutilans*	25 nM	Yang et al., 2013
	Hainantoxin-IV	*Ornithoctonus hainana*	21 nM	Liu et al., 2003
	μ-TRTx-Pn3a	*Pamphobeteus nigricolor*	0.9 nM	Deuis et al., 2017
	GrTx1	*Grammostola rosea*	0.37 μM	Redaelli et al., 2010
	GVIIJ_SSG_	*Conus geographus*	41 nM	Gajewiak et al., 2014
	Huwentoxin-IV	*Haplopelma schmidti*	26 nM; 0.4 nM	Xiao et al., 2008; Rahnama et al., 2017
Na_v_1.8	ProTx-I	*Thrixopelma pruriens*	27 nM	Middleton et al., 2002
	MrVIB (μO-Conotoxin)	*Conus marmoreus*	102 nM	Ekberg et al., 2006
	MfVIA (μO-Conotoxin)	*Conus magnificus*	529 nM	Vetter et al., 2012
	HSTX-I	*Haemadipsa sylvestris*	2.44 μM	Wang et al., 2018
Na_v_1.9	HSTX-I	*Haemadipsa sylvestris*	3.30 μM	Wang et al., 2018
Ca_v_1.2	Calciseptine	*Dendroaspis polylepis polylepis*	430 nM	de Weille et al., 1991
Ca_v_2.1	ω-conotoxin CVIB	*Conus catus*	7.7 nM	Lewis et al., 2000
	ω-conotoxin CVIC	*Conus catus*	7.6 nM	Lewis et al., 2000
	ω-conotoxin MVIIC	*Conus magus*	7 nM	Lewis et al., 2000
	ω-agatoxin IVA	*Agelenopsis aperta*	0.1 μM	Mintz et al., 1992
	ω-grammotoxin SIA	*Grammostola rosea*	50 nM	Lampe et al., 1993; McDonough et al., 1997
Ca_v_2.2	ω-agatoxin IIA	*Agelenopsis aperta*	10 nM	Bindokas and Adams, 1989; Adams et al., 1990
	ω-agatoxin IIIA	*Agelenopsis aperta*	1.4 nM	Ertel et al., 1994; Olivera et al., 1994
	ω-agatoxin IIIB	*Agelenopsis aperta*	140 nM	Ertel et al., 1994; Yan and Adams, 2000
	ω-agatoxin IIID	*Agelenopsis aperta*	35 nM	Ertel et al., 1994
	ω-ctenitoxin-Pn3a/Neurotoxin Tx3–4	*Phoneutria nigriventer*	50 pM	Cordeiro Mdo et al., 1993
	ω-conotoxin CVIA	*Conus catus*	0.6 nM	Lewis et al., 2000
	ω-conotoxin CVIB	*Conus catus*	7.7 nM	Lewis et al., 2000
	ω-conotoxin CVIC	*Conus catus*	7.6 nM	Lewis et al., 2000
	ω-conotoxin CVID	*Conus catus*	0.07 nM	Lewis et al., 2000
	ω-conotoxin MVIIA	*Conus magus*	0.055 nM	Lewis et al., 2000
	ω-conotoxin GVIA	*Conus geographus*	0.04 nM	Olivera et al., 1984; Lewis et al., 2000
Ca_v_2.3	SNX482	*Hysterocrates gigas*	15–30 nM	Newcomb et al., 1998
Ca_v_3.1	Kurtoxin	*Parabuthus transvaalicus*	15–50 nM	Chuang et al., 1998; Sidach and Mintz, 2002
	ProTx1	*Thrixopelma pruriens*	200 nM	Ohkubo et al., 2010
Ca_v_3.2	Kurtoxin	*Parabuthus transvaalicus*	25–50 nM	Chuang et al., 1998; Sidach and Mintz, 2002


## Venom Peptides Targeting Potassium Channels

Potassium ion channels are of high therapeutic value due to their broad and active presence in a variety of human tissue. To date, numerous disease conditions in neuronal, cardiac, immune, and endocrine systems have been reported to be directly associated with malfunction of potassium channels. Potassium channels are categorized into four families: two transmembrane (TM) Kir channels, four TM, two pore-domain K2P channels, and six TM K_v_ and K_Ca_ channels (Chuan et al., 2013). Here, we discuss the Kir, K_v_ and K_Ca_ channels. The K2P family of channels contribute to voltage-independent “leak” K^+^ current, and are structurally different from other classes of K^+^ channels in that they assemble as ‘dimer of dimers’ (Goldstein et al., 2005). No venom-derived peptide toxins have been reported for K2P channels yet (McQueen, 2017).

Inwardly rectifying potassium (Kir) channels were first described in 1949 in frog skeletal muscles (Katz, 1949), however, they were not cloned and isolated until 1993 (Ho et al., 1993; Kubo et al., 1993). As the name suggests, Kir channels inwardly rectify outward K^+^ current, allowing extracellular K^+^ to readily flow into the cells. The unique molecular mechanism is due to the intracellular binding of Mg^2+^ and polyamines (Lu, 2004). Kir channels are homo- or hetero- tetrameric structures assembled from four Kir subunits, containing two TM segments separated by a selectivity filter region (Whorton and MacKinnon, 2011; Li et al., 2017). Structural, functional and pathophysiological details of four specific types of Kir channels have been detailed elsewhere (Hibino et al., 2010).

The peptides that show high affinity toward Kir channels (IC_50_ < 0.5 μM) are scorpion toxin ChTx2 (α-KTx1.2), snake toxin δ-dendrotoxin (δ-DTX), and honey bee toxin Tertiapin (TPN) (Lu and MacKinnon, 1997; Imredy et al., 1998; Jin and Lu, 1998; Doupnik, 2017). Like many other venom toxins, these three molecules are rich in cysteine and positively charged residues. Computational simulation and docking studies have hypothesized binding mechanisms of these toxins (Li et al., 2016). Positively charged residues from toxin come into close contact with negatively charged residues on channel pore region, strengthening electrostatic interactions between the two. Hydrophobic forces between aliphatic residues also count into binding affinity.

TPN and TPN_M13Q_ are considered the most potent inhibitors. TPN binds to Kir1.1 and Kir3.1/3.4 at 2-8 nM (EC_50_), thus being an ideal tool for investigations of Kir channels’ functional and pharmacological properties (Dobrev et al., 2005; Walsh, 2011). TPN has shown potential therapeutic use in a canine model, treating atrial fibrillation, without causing ventricle arrhythmia (Hashimoto et al., 2006). More recently, TPN, together with sodium channel blockers, has been shown to have synergistic effects in preventing atrial fibrillation and prolonging atrial effective refractory period. The combination formula has been patented for medication manufacturing by Gilead Sciences.

Voltage-gated potassium (K_v_) channels tightly control membrane permeability of K^+^ by sensing voltage change, thereby playing a key role in regulating action potential and propagating electrical signals in excitable cells (Yellen, 2002). In non-excitable cells, K_v_ channels modulate cellular metabolism and facilitate downstream signaling cascade; for example, K_v_1.3 in T lymphocytes (Cahalan and Chandy, 2009). 40 K_v_ channels in 12 subfamilies have been found and many extensively studied (Alexander et al., 2017). K_v_ channels are homo- or hetero- tetramers, made up of four subunits each consisting of six TM helices. Voltage sensing domain (VSD) (S1–S4) is connected to pore domain (S5–S6) through S4–S5 intracellular loop, driving the pore to open or close (Long et al., 2005).

Research on venom peptide modulators of K_v_ channels started in 1980s, and to date more than 200 peptides with inhibitory effect on K_v_ channels have been identified (Carbone et al., 1982; Kuzmenkov et al., 2016). These polypeptides usually bind to K_v_ channels in two unique mechanisms. The pore blockers sit in the shallow vestibule at extracellular pore region, while the gating modifiers bind to the so-called “paddle motif” of the VSD accessible from the extracellular side.

Scorpion toxin charybdotoxin (ChTx) was one of the earliest venom toxins used as an important research tool to understand K_v_ channel subunit stoichiometry (MacKinnon, 1991), auxiliary beta subunits (Garcia et al., 1995), as well as its overall architecture (Hidalgo and MacKinnon, 1995).

Sea anemone toxin ShK blocks K_v_ channels at nanomolar to sub-nanomolar potency (Castañeda et al., 1995; Kalman et al., 1998). ShK and its analogs are blockers of the K_v_ channel pore. They bind to all four subunits in the channel tetramer by two key interactions within the external vestibule – Lys22 occludes the channel pore like a “cork in a bottle,” and Tyr23, together with Lys22, forms a “functional dyad” required for channel block. Many K^+^ channel-blocking peptides exhibit similar blocking mechanism, consisting of a dyad of lysine and neighboring aromatic/aliphatic residue (Chang et al., 2018). With the goal of developing a highly selective K_v_1.3 inhibitor, nearly 10 years of effort was made to re-engineer the native ShK. In 2006, a stable analog, ShK-186 demonstrated specific binding to K_v_1.3 at 69 pM, which is 100-fold selective to other K_v_ channels (Chi et al., 2012). ShK-186 (Dalazatide), now being developed by Kineta, has passed phase I clinical trials It is the only venom-derived peptide blocking K^+^ channels that is being developed as a therapeutic (Tarcha et al., 2012, Tarcha et al., 2017).

The hERG channel (or K_v_11.1) plays a crucial role in the cardiac action potential by repolarizing IKr current, the rapid component of the delayed rectifier potassium current. While selective K_v_11.1-blockers are available (e.g., BeKm-1 from scorpion *Mesobuthus eupeus*) (Korolkova et al., 2001), it warrants special attention as many drugs/peptides intended for other targets, can exhibit non-selective binding to it, with potentially fatal consequences. Inhibition of hERG by drugs can lead to lengthening of the electrocardiographic QT interval, while hERG channel activators can cause drug-induced short QT syndrome. Both cases can lead to potentially fatal arrhythmias. Hence, FDA guidelines recommend that all drugs that are intended for human use be evaluated for anti-hERG activity (Vandenberg et al., 2012).

Calcium (Ca^2+^)-activated potassium channels (K_Ca_) are broadly divided into three subtypes based on their single channel conductance - big conductance (BK_Ca_), intermediate conductance (IK_Ca_) and small conductance (SK_Ca_). While the BK_Ca_ channels are activated by both voltage and increase in cytosolic Ca^2+^, the IK_Ca_ and SK_Ca_ channels are activated exclusively by the latter. Like Kir and K_v_ channels, the K_Ca_ channels are tetramers made up of four α subunits. BK_Ca_ requires additional regulatory subunits, and is made up of 6/7 TM segments, while SK_Ca_ and IK_Ca_ contain 6 TM segments, with a calmodulin molecule bound to each subunit, serving as the Ca^2+^ sensor. One of the first peptide toxins that were found to inhibit K^+^ channels included apamin (derived from bee venom) and charybdotoxin (ChTX, derived from the scorpion venom) (Hugues et al., 1982; Rauer et al., 2000). Apamin blocks SK channels (K_Ca_2), and served as a primary pharmacological tool to distinguish between K_Ca_2 channels and K_Ca_1.1/K_Ca_3.1. ChTX inhibits both K_Ca_ channels (K_Ca_1.1 and K_Ca_3.1) and K_v_ channels (K_v_1.2, K_v_1.3, and K_v_1.6). Another scorpion toxin iberiotoxin is selective for BK channel (K_Ca_1.1) (Candia et al., 1992).

## Venom Peptides Targeting Voltage-Gated Sodium Channels

Voltage-gated sodium (Na_v_) channels are present in the membranes of most excitable cells and are responsible for initiation and propagation of action potentials. Studies elucidating details of ion selectivity, hypothesizing the Na_v_ pore diameter and binding mechanism of sodium-channel acting local anesthetics and related drugs, were bolstered by the availability of ion channel toxins, like the alkaloids tetrodotoxin (TTX) and saxitoxin (STX) (Hille, 1971, 1975, 1977; Armstrong et al., 1973). Studies to isolate and purify the Na_v_ channel protein were pioneered by William Catterall and co-workers using, besides TTX and STX, scorpion toxin (ScTx) neuropeptides (Agnew et al., 1978; Beneski and Catterall, 1980; Hartshorne and Catterall, 1981).

Na_v_ channels are divided into nine subtypes (Na_v_1.1–Na_v_1.9) based on their sequence, TTX binding and tissue expression. The 250 kDa channel-forming α-subunits are pseudo-tetrameric, wherein a single polypeptide chain folds into four homologous, non-identical domains (DI–IV), each containing six TM segments (S1–S6). The S5–S6 segments from all four domains form the central ion pore, while the S1–S4 segments in each domain form the VSD. A single channel is composed of one pore-forming α subunit, which may be associated with either one or two β subunits. The α subunit is functional on its own, and forms the core of the channel.

The venom of various animals contain toxins that target Na_v_ channels to attack the neuromuscular systems of their adversaries and prey. Toxins that modulate Na_v_ channel function generally do so in two ways – either by blocking the flow of Na^+^ ions through the pore, or by modifying the gating mechanisms.

One of the best studied pore blockers for Na_v_ channels are the μ-conotoxin peptides from cone snails. Conotoxins are disulfide-rich peptides that are isolated from the venom of cone snails (genus Conus). Venom derived from cone snails is a treasure trove of peptide toxins for different ion channels and other receptor proteins (Olivera et al., 1985, 1990). M-conotoxins demonstrate the best binding with the skeletal muscle isoform of Na_v_ channel, Na_v_1.4, with variable binding to other isoforms. These variations in targeting selectivity and affinity of each peptide for the different Na_v_ isoforms constitute an important tool for distinguishing between different isoforms (Zhang et al., 2013). On the other hand are toxin peptides that modify Na_v_ channel gating by interacting with the voltage sensors. Various classes of conotoxins interact with the voltage sensors of Na_v_ channels and influence their gating properties. Δ-conotoxins are ubiquitously expressed in a range of cone snail venoms and inhibit fast inactivation of channels. While the μ-conotoxins are pore-blocking peptides, the μO-conotoxins are gating modifiers that target the voltage sensors and inhibit channel opening (Daly et al., 2004; Zorn et al., 2006; Leipold et al., 2007). MO-conotoxins were evaluated for their pain-relieving activity and found to be anti-nociceptive in animal models of pain (Teichert et al., 2012).

Several spider toxins are in pre-clinical development stage as antagonists of Na_v_1.7, an attractive target for development of non-opiod pain medication. Protoxin-II (ProTX-II), derived from the tarantula *Thrixopelma pruriens*, inhibits channel activation by shifting to positive potentials the voltage dependence of channel activation. Using ProTX-II as a scaffold, a highly potent and selective Na_v_1.7 blocking peptide (JNJ63955918) has been developed, the effect of which mirrors features of the Na_v_1.7-null phenotype (Flinspach et al., 2017). Another venom peptide, huwentoxin IV, is derived from the Chinese bird-eating spider *Selenocosmia huwena* (Peng et al., 2002). This peptide preferentially inhibits Na_v_1.7 by binding one of the four VSDs of the channel, making it more selective as compared to the local anesthetics that bind the conserved channel pore (Ragsdale et al., 1996; Xiao et al., 2008, 2011). Various mutational studies led to a triple mutant of huwentoxin IV (E1G, E4G, and Y33W) being developed with a very high potency toward Na_v_1.7 blocking (Revell et al., 2013).

Na_v_1.5 is expressed mainly in cardiac muscle, where it mediates fast depolarization phase of the cardiac action potential and is a target for class I anti-arrhythmic agents. Jingzhaotoxin-III (from the Chinese tarantula *Chilobrachys jingzhao*) selectively inhibits the activation of Na_v_1.5 in heart cells (IC50 ∼ 350 nM), but not Na_v_ neuronal subtypes (Rong et al., 2011).

Sea anemones are another source of Na_v_-targeting peptides. Some key toxins are ATX-II (from *Anemonia sulcata*), AFT-II (from *Anthopleura fuscoviridis*) and Bc-III (from *Bunodosoma caissarum*). ATX-II strongly affects Na_v_1.1 and Na_v_1.2, while AFT-II affects Na_v_1.4 and Na_v_1.5. Given that these two differ in a single amino acid (ATX-II→K36A→AFTII), indicates that the lysine at position 36 is important for the very strong effects of ATX-II on Na_v_1.1/2 channels (Oliveira et al., 2004; Moran et al., 2009).

## Venom Peptides Targeting Voltage-Gated Calcium Channels

Voltage-gated calcium channels (Ca_v_) facilitate cellular calcium influx in response to membrane depolarization. They regulate hormone secretion, neurotransmitter release, propagation of cardiac action potential, muscle contraction and gene expression in different cell types (Catterall, 2011).

Similar to the Na_v_ channels, the α1 subunit of Ca_v_ channels is organized in four homologous domains (I–IV), each containing six TM segments (S1–S6). The S1–S4 segments constitute the voltage sensor, while S5–S6 constitute the pore. Auxiliary subunits usually associate with α1, regulating channel expression and function. Ca_v_ channels are grouped into various types based on their electrophysiological and pharmacological properties and tissue distribution – L-type (Ca_v_1 subfamily: Ca_v_1.1-Ca_v_1.4); P/Q-, N-, and R-types (Ca_v_2.1, Ca_v_2.2 and Ca_v_2.3, respectively) and T-type (Ca_v_3 subfamily: Ca_v_3.1-Ca_v_3.3). Venom toxins have played a vital role in the discovery of, and in deciphering the structure and function of, many Ca_v_ channels. Chief among them are the ω-conotoxins and ω-agatoxins.

Ω-conotoxins are ∼24–30 residues in length and contain three intramolecular disulfide bonds. They target Ca_v_ channels via blocking the ion pore. Ω-conotoxin GVIA, from the venom of *Conus geographus*, was the first of the ω-conotoxins to be isolated and characterized (Kerr and Yoshikami, 1984; Olivera et al., 1985). Studies with GVIA showed inhibition of Ca^2+^ entry (voltage-activated), and GVIA was a powerful probe to explore the presynaptic terminal, linking Ca_v_ (N-type) channels to neurotransmitter release and synaptic transmission (Kerr and Yoshikami, 1984; Olivera et al., 1984). Molecular identity of the N-type and L-type channel subunit composition was determined using GVIA binding (Williams et al., 1992).

Subsequent to GVIA, many other ω-conotoxins were identified. One of the most prominent ones is MVIIA, from the Magician’s cone snail, *Conus magus* (Olivera et al., 1987), which was tested and developed as a therapeutic agent against pain. Ziconotide (Prialt^®^) has been clinically approved for the treatment of severe chronic pain associated with cancer and neuropathies, and is currently the only venom peptide drug targeting a voltage-gated ion channel (Ca_v_2.2) that is in clinical use (Miljanich, 2004). A more selective ω-conotoxin, CVID, was isolated from *Conus catus* (Lewis et al., 2000), and was being developed as leconotide for pain treatment. However, it failed clinical trials due to adverse side-effects (Kolosov et al., 2010).

Spider toxin ω-agatoxin IVA, a gating modifier toxin isolated from *Agelenopsis aperta*, specifically targets P/Q-type channels (Pringos et al., 2011), and was used to study the channel subunit composition (McEnery et al., 1991; Witcher et al., 1995).

## Concluding Remarks

Given that there are many species whose toxic venom are yet to be fully explored, the collection of venom-derived peptides to be discovered is immense. Also, with the advent of technology in drug design, based on currently available toxin peptides, new drugs will be developed into more stable and selective biologics. While venomous species developed toxins to incapacitate prey and predators, and envenomation is a public health hazard for us humans, the toxins have proven to be an excellent source of research and therapeutic tools.

## Author Contributions

All authors listed have made a substantial, direct and intellectual contribution to the work, and approved it for publication.

## Conflict of Interest Statement

The authors declare that the research was conducted in the absence of any commercial or financial relationships that could be construed as a potential conflict of interest.
